# Canakinumab treatment in patients with active recurrent or chronic TNF-receptor associated syndrome (TRAPS): Efficacy and safety results from a proof of concept study

**DOI:** 10.1186/1546-0096-13-S1-O59

**Published:** 2015-09-28

**Authors:** H Lachmann, M Cattalini, L Obici, R Barcellona, A Speziale, Y Joubert, G Junge, M Gattorno

**Affiliations:** 1 University College London, London, UK; 2 Pediatric Clinic, University of Brescia, Brescia, Italy; 3 Policlinico S. Matteo, Pavia, Italy; 4 Hospital Sciacca, Sciacca, Italy; 5 Novartis Pharma AG, Basel, Switzerland; 6 G Gaslini Institute, Genova, Italy

## Introduction

Tumor necrosis factor receptor-1 associated periodic syndrome (TRAPS) is a periodic fever syndrome, characterized by recurrent fever attacks associated with rashes, musculoskeletal and abdominal pain, and periorbital edema. In few patients TNF inhibitors have been shown to be effective, however, their efficacy tends to decrease over time [1-4]. Anti-IL-1 treatments have also been used in an effort to provide long term control of the inflammatory manifestations.

## Objectives

The main objective was to determine whether canakinumab (CAN) induced complete or almost complete response in patients with active TRAPS at Day 15, as defined by Physician's Global Assessment (PGA). Evolution of C-reactive protein (CRP) and serum amyloid A (SAA), relapse rate and time to remission were additional measures. An additional objective was to determine the appropriate dosing for further development of CAN treatment in TRAPS patients.

## Patients and methods

This was an open-label, single treatment arm, efficacy and safety study of monthly CAN 150 mg (2 mg/kg for patient ≤40 kg) SC in patients with active recurrent or chronic TRAPS [NCT01242813]. Patients were treated for 4-months with a maximum 5-month follow-up period. The initial follow-up period was followed by a maximum 24-month long-term treatment period. Here we report the efficacy and safety results of the completed study.

## Results

A total of 20 patients were exposed to study medication, out of which 18 (90%) patients completed the study. At Day 15, complete response or almost complete response was achieved in 18 patients [90%; 95% CI: 75.1, 99.9], while 20 (100%) and 12 (60%) patients had clinical and serological remission, respectively. Already at Day 8, 16 patients (80%) achieved a complete or almost complete response, while 18 (90%) and 7 (35%) patients had clinical and serological remission, respectively. A total of 60% of patients experienced study drug-related AEs, most commonly upper respiratory tract infections. Seven patients (35%) experienced SAEs, none of which were related to study drug. Furthermore, there were no deaths during the study.

## Conclusions

Canakinumab was effective in rapidly improving clinical signs and symptoms of TRAPS, whilst normalizing serological inflammatory markers and providing sustained disease control. The safety profile was consistent with previous canakinumab studies in other indications. These data support the ongoing development of canakinumab in this therapeutic area.
